# Prevalence and Antimicrobial-Resistant Features of *Shigella* Species in East Africa from 2015–2022: A Systematic Review and Meta-Analysis

**DOI:** 10.1155/2023/8277976

**Published:** 2023-09-02

**Authors:** Basha Ayele, Getenet Beyene, Mekdelawit Alemayehu, Aman Dekebo, Zeleke Mekonnen, Gashaw Nigussie

**Affiliations:** ^1^Department of Medical Laboratory Science, College of Health Science and Medicine, Dilla University, P.O. Box: 419, Dilla, Ethiopia; ^2^School of Medical Laboratory Sciences, Institution of Health, Jimma University, Jimma, Ethiopia; ^3^Armauer Hansen Research Institute, P.O. Box 1005, Addis Ababa, Ethiopia; ^4^Department of Applied Chemistry, Adama Science and Technology University, P.O. Box 1888, Adama, Ethiopia; ^5^Institute of Pharmaceutical Sciences, Adama Science and Technology University, P.O. Box 1888, Adama, Ethiopia

## Abstract

**Background:**

Shigellosis is the most common cause of epidemic dysentery found worldwide, particularly in developing countries, where it causes infant diarrhea and mortality. The prevalence of *Shigella* species resistant to commonly used antimicrobial drugs has steadily increased. The purpose of this review is to describe the prevalence and antimicrobial resistance (AMR) characteristics of *Shigella* species in East Africa between 2015 and 2022.

**Methods:**

Studies were identified using a computerized search of Medline/PubMed, Google Scholar, and Web of Science databases, with a detailed search strategy and cross-checking of reference lists for studies published between 2015 and 2022. Articles presenting data on prevalence and AMR, accessibility of the full-length article, and publication dates between 2015 and 2022 were the eligibility criteria for inclusion in the review. Original research reports written in English were considered. The heterogeneities of the studies were examined, and a meta-analysis was performed to estimate the pooled prevalence and AMR using a random effects model.

**Results:**

The pooled prevalence of *Shigella* species in East Africa was 6.2% (95% CI −0.20–12.60), according to an analysis of 22 studies. *Shigella* species prevalence was 4.0% in Ethiopia, 14.6% in Kenya, 0.7% in Sudan, 5.2% in South Sudan, and 20.6% in Somalia. The association of *Shigella* infection significantly varied among the countries (*p* = 0.01). Among the antibiotics tested, most *Shigella* isolates were susceptible to ciprofloxacin, norfloxacin, nalidixic acid, and ceftriaxone. Despite the fact that the reports varied in study sites and time, *Shigella* species were resistant to tetracycline, ampicillin, amoxicillin, chloramphenicol, and co-trimoxazole.

**Conclusion:**

The pooled estimate indicates high burden of *Shigella* infection in East Africa, as well as a high proportion of drug resistance pattern to tetracycline, ampicillin, chloramphenicol, and amoxicillin. Therefore, initiating and scale-up of performing drug susceptibility test for each shigellosis case need to be considered and strengthened.

## 1. Background

Shigellosis is caused by the ingestion of bacteria of the genus *Shigella*. Kiyoshi Shiga discovered the bacterium in the stool of patients suffering from severe bloody diarrhea in Japan in 1897 [1]. *Shigella* is a Gram-negative bacterium that causes diarrhea and dysentery in humans. There are four species of *Shigella* based upon serological and biochemical characteristics: *Shigella dysenteriae* (*S. dysenteriae*), *S. flexneri*, *S. boydii*, and *S. sonnei* [2]. Serogroup A (*S. dysenteriae*) has 15 serotypes and 2 provisional serotypes [1, 3], serogroup B (*S. flexneri*) has 6 serotypes and 16 subserotypes, serogroup C (*S. boydii*) has 20 serotypes, and serogroup D (*S. sonnei*) has only 1 serotype [4].

The burden of diarrheal disease is the greatest in developing countries with poor sanitation, insufficient hygiene, contaminated drinking water, and poorer overall health and nutritional status [5]. In comparison to other causes of gastroenteritis, it is a highly infectious microorganism because only 10 bacilli of microorganisms are required to cause infection [6]. Fever, fatigue, anorexia, and malaise are common symptoms of the disease. Some patients suffer from mild to severe dysentery, with systemic complications such as electrolyte imbalance, seizures, and hemolytic uremic syndrome [7].

Shigellosis is the leading cause of infant diarrhea and mortality in developing countries [2]. The domination of *S*. *flexneri* is observed in Africa and Asia, whereas *S. sonnei*, the most dominant in South America, is primarily isolated in one study in Ethiopia [8]. This may give a clue to the scientific world about the migration and movement of strains from one region to the other. Such variations could be attributed to differences in disease epidemiology between study sites. The prevalence of *Shigella* species reports varies across studies, which may be due to location difference, study methods, and techniques used [7]. In developing countries, it is difficult to evaluate the burden of *Shigella* infection because of the limited scope of studies and lack of coordinated epidemiological surveillance systems. In addition, under-reporting of cases and the presence of other diseases considered to be of high priority may have overshadowed the problem of shigellosis.

The emergence of multidrug-resistant (MDR) *Shigella* strains and the development of the disease state have complicated case management [9]. An increment of MDR to shigellosis among several serotypes of *Shigella* species isolated from acute diarrheal patients [3, 10]. Regardless of the serogroup or serotype, the majority of the strains carried similar gene-encoding resistance to specific antimicrobials. This drug resistance emergency necessitates the prudent use of effective drugs and emphasizes the need for alternative drugs to treat infections caused by resistant strains. The pattern of AMR varies by location and between two regions within the same location [1]. The emergence of MDR to available antimicrobials, the lack of reliable vaccination, the disease's increasing occurrence worldwide, and the disease's high incidence in high-risk populations all provide compelling reasons to conduct this review. Despite the high prevalence of shigellosis, summary data on *Shigella* species in East Africa are scarce. Therefore, this reviewer focused on prevalence and antimicrobial-resistant features of *Shigella* species in East Africa from 2015 to 2022.

## 2. Methods

### 2.1. Search Strategy and Eligibility Criteria

**Figure 1:**
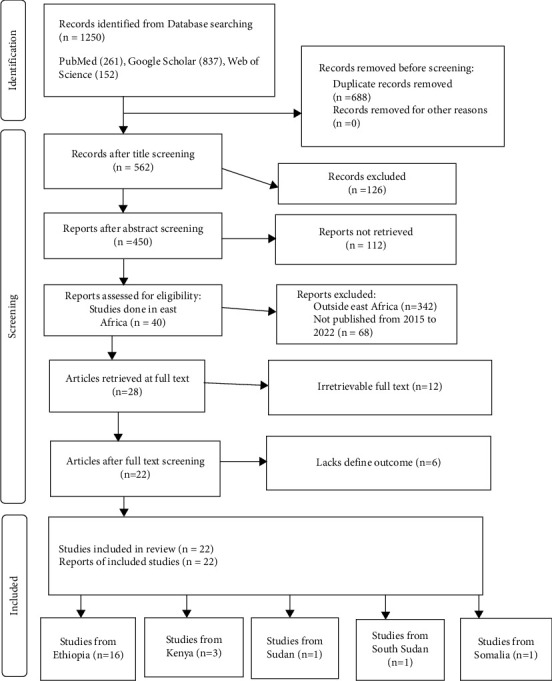
Chosen recording items for systematic reviews and meta-analysis flowchart for the selection of studies incorporated in the systematic review and meta-analysis.

Original research that provided information on the prevalence and AMR of *Shigella* species was used to review published publications. Studies were identified through a computerized search using databases of Medline/PubMed, Google Scholar, and Web of Science which were included with a detailed search strategy and cross-checking of reference lists for studies published from 2015 to 2022 in East Africa. The criteria for studies' eligibility were in accordance with study sites and the PRISMA statement' outcome approach. Studies in Ethiopia, Kenya, Sudan, South Sudan, and Somalia were reviewed; however, due to requirements for article inclusion, the remaining East African nations were not provided. The study outcome search concentrated on the prevalence of *Shigella* species and the AMR on the patterns of *Shigella* species' susceptibility to antibiotics. Articles containing prevalence and AMR statistics, full-text primary studies published in English, and publication dates between 2015 and 2020 were required for inclusion in the review (Figure 1). Papers that lacked the necessary details as well as unpublished theses and dissertations were not included. After completing the searches, all the retrieved records were downloaded and stored in a single library in EndNote 20 (Thompson Reuters).

### 2.2. Data Abstraction for Analysis

In cases where there was insufficient detail supplied, the complete article was reviewed to determine whether it should be included or excluded. To choose which studies to include in the narrative synthesis, the reviewer (BA) deleted duplicates from the EndNote library both automatically and manually. The remaining records were then screened by the same reviewer, first based on the title and then based on the abstract. The shortlisted articles were then retrieved in full text to determine their suitability for final inclusion. The extraction sheet format was piloted in 5% of the studies chosen randomly before being deployed. The article was included based on a full-text analysis. Because of differences in the study, publications were extensively evaluated when data were extracted. The reviewer (BA) was contacted (at least three times) through email to provide clarification where necessary information was needed but lacking. The heterogeneities of the studies were examined. Using comprehensive meta-analysis, overall pooled prevalence and AMR of *Shigella* species were estimated by the random effects model. Analysis with a 95% confidence interval (CI), *P* ≤ 0.05, was considered as statistically significant.

## 3. Results

### 3.1. Prevalence of *Shigella* Species

**Figure 2:**
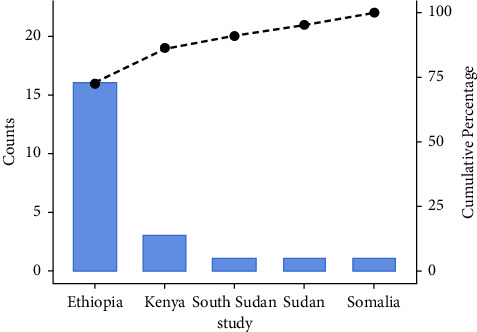
Study of *Shigella* species by region.

**Figure 3:**
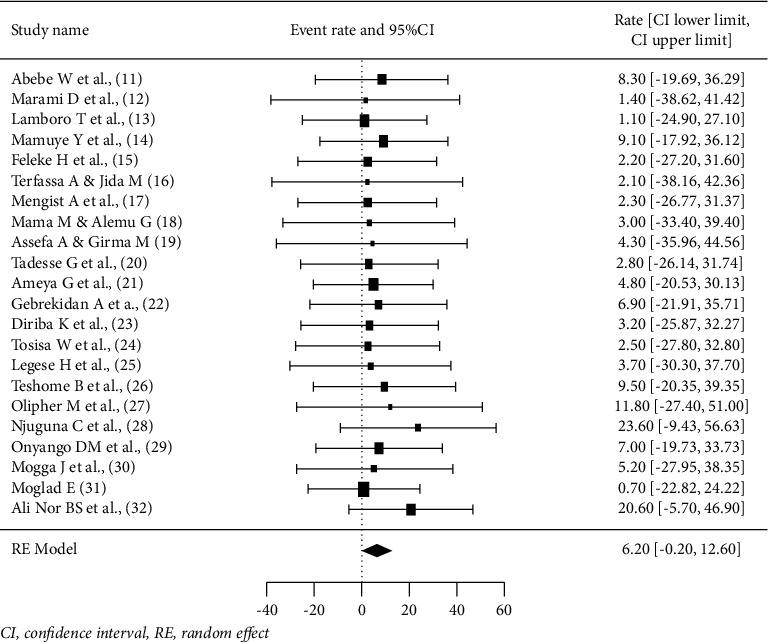
Forest plot for the prevalence of *Shigella* species in East Africa.

**Figure 4:**
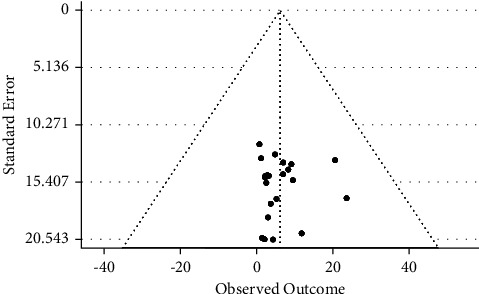
Funnel plot for the prevalence of *Shigella* species in East Africa.

22 studies with 5694 samples were included in our review of 450 titles and abstracts, including 16 research from Ethiopia, 3 studies from Kenya, and 3 studies each from South Sudan, Sudan, and Somalia (Figure 2). The included studies' enrollment periods spanned 2015 to 2022. The reviewed studies included 144 sample sizes with the smallest and 422 samples with the largest in Sudan and Ethiopia, respectively (Table 1). Majority of the studies were performed on the genus level. Seven studies were performed on asymptomatic food handlers. Seven studies were performed under five children and the remaining studies were included without age restriction patients with diarrhea and nondiarrheic in this review. Children and diarrheic patients were more associated with shigellosis. Of the adult subject studies, males were more associated with *Shigella* infection. The overall prevalence of Shigella species was in the range of 0.7–23.6% with *S. flexneri*being the most frequently isolated which revealed this species as predominant in the etiology of shigellosis followed by *S. dysenteriae*, *S. boydii*, and *S. sonnei*from the serogroup studies in East Africa. The analysis of 22 studies, according to the DerSimonian–Laird random-effects model, revealed that the pooled prevalence of *Shigella* species in East Africa was 6.2% (95% CI −0.20–12.60) (Figures 3 and 4). Pooled prevalence of *Shigella* species significantly varied among the countries (*p* = 0.01), with 4.0% in Ethiopia, 14.6% in Kenya, 0.7% in Sudan, 5.2% in South Sudan, and 20.6% in Somalia. In most of the investigations performed in Ethiopia, *Shigella* infection rates did not change significantly (*p* > 0.05) (Table 2 and Figure 3).

### 3.2. Antimicrobial Resistance of S*higella* Species

**Figure 5:**
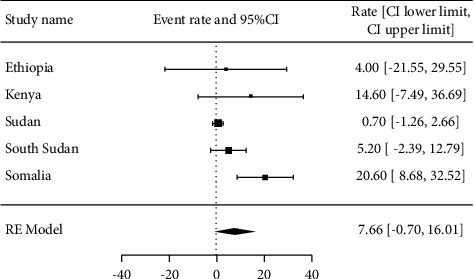
Forest plot for the prevalence of antibiotics resistance in East Africa.

**Figure 6:**
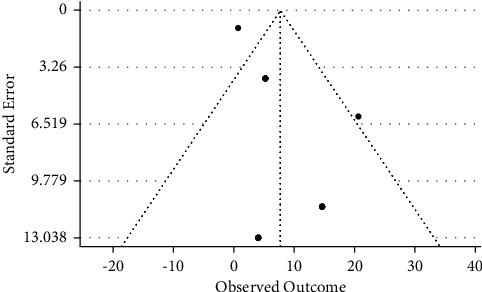
Funnel plot for the prevalence of antibiotics resistance in East Africa.

Most *Shigella* species isolates tested sensitive for ciprofloxacin, norfloxacin, nalidixic acid, and ceftriaxone. Even though the reports varied in research locations and times, *Shigella* species were resistant to tetracycline, ampicillin, amoxicillin, chloramphenicol, and co-trimoxazole. The vast majority of investigations demonstrated the existence of general MDR patterns. The overall pooled prevalence of antibiotics resistant to *Shigella* species was 7.7% in East Africa (Figures 5 and 6). The pooled resistance of *Shigella* species was 58.3% for ampicillin, 46% for tetracycline, 33.2% for chloramphenicol, 30.4% for amoxicillin, and 23.7% for co-trimoxazole. Comparatively, low resistance pattern was reported in ciprofloxacin (11.7%), gentamicin (9.3%), nalidixic acid (8.0%), ceftriaxone (7.1%), and norfloxacin (1.6%) (Table 2).

## 4. Discussion

A lot of studies were conducted in different parts of the world even if those studies were performed on the genus level of *Shigella*. This review study described prevalence and AMR patterns of *Shigella* species in East Africa from 2015 to 2022. In this review, children and diarrheic patients were more associated with shigellosis. This might be that the children at this age are naturally taking contaminated soils, food, and water into their mouth and may acquire disease-causing microbes including *Shigella* species [19, 33]. In the review study in Ethiopia [34], the pooled prevalence of shigellosis in children was 7.0%, while in adult population, it was 2.2%. This confirms that *Shigella* causes diarrheal morbidity among infants and young children than adults. Children who drank from unimproved water sources, untrimmed finger nails, and that which was served by parents who did not wash their hands before meal were more likely to be exposed to *Shigella* infection [33]. Unimmunized children also had higher infection risk than those who were immunized to different infectious diseases [20, 21]. Due to the ability of the bacteria to invade and replicate in cells lining the colon and rectum, patients with bloody diarrhea and mixed (mucus and blood) were more positive to *Shigella* species [22]. This study reviewed that males were more associated with *Shigella* species on the adult subject studies. This might be that males travel more to the different regions and seek diagnosis [35–37]. In addition, this could be the study population by itself, as Chattaway et al. stated that a high male to female ratio with 97% of cases being adult males in the cluster [38].

This review determined the pooled prevalence of *Shigella* species in East Africa using 22 studies. According to the results of this review, the pooled prevalence was 6.2%. This finding is comparable with 6.6% *Shigella* prevalence in the systematic review among US military and similar populations [39] and meta-analysis in Ethiopia [34]. Prevalence of *Shigella* species among East African countries was also calculated; hence, a higher prevalence of *Shigella* species (20.6%) was reported in Somalia, which was nearly 5 and 29 times higher than the findings from Ethiopia (4.0%) and Sudan (0.7%), even though the studies conducted and included in this review from this country was only one study. The variations in prevalence estimates may be due to differences in the study populations, year of study, and number of studies. As a study confirmed that the prevalence of *Shigella* species reports varies in different regions and time [7], the decreased in prevalence might be due to decrease in poverty, increase quality of life, rise of awareness on sanitation and hygiene, and prevention and control strategy of communicable diseases through deploying of health extension workers at community level across the country.

Based on the data obtained from 22 published articles, *Shigella* species showed high resistance to tetracycline, ampicillin, chloramphenicol, and amoxicillin. This finding is in line with the study performed on AMR [34, 40]. Commonly in East Africa, the drug of choice on shigellosis treatment is norfloxacin, ciprofloxacin, and ceftriaxone for adults. However, this review showed that slightly high resistance was reported on norfloxacin, ciprofloxacin, and ceftriaxone. Furthermore, the occurrences of MDR of *Shigella* isolates were reported high. This increment may be due to mobile genetic units (including plasmids, gene cassettes in integrons, and transposons), inadequate access to effective drugs, unregulated dispensing, truncated antimicrobial therapy, medication sharing, counterfeit drugs, bacterial evolution, climate changes, lack of medical practitioner with proper training, poor quality, and unhygienic sanitary conditions [37]. Except a few studies, all are performed on the genus level. High rates of resistance against multiple antimicrobials were also observed in most of the isolates. The most resistant isolates from *Shigella* species were *S. flexneri*, which showed 87.5% resistance to ampicillin, 75% to tetracycline, and 62.5% to ciprofloxacin. *S. dysenteriae* was the second most resistant bacteria, which showed 80% resistance to chloramphenicol and tetracycline, 70% to ampicillin, and 60% to ciprofloxacin [26]. Another study conducted in Somalia [32] showed the highest resistance to ceftriaxone occurred among *S. sonnei* (66.7%) serogroup, followed by *S. dysenteriae* type 1 (40%) and *S. flexneri* (38.5%). In this review, included studies primarily used stool culture for *Shigella* identification. This estimate appears to be a less sensitive method than molecular methods and may underestimate the actual occurrence of *Shigella* species [36].

## 5. Conclusion

This review study suggests that the current treatment mechanism might not be addressing the full burden of *Shigella*-associated mortality in East Africa. The pooled estimate provides high burden of *Shigella* infection and its high proportion of drug resistance pattern to tetracycline, ampicillin, chloramphenicol, and amoxicillin in East Africa. Clinicians should continue to aggressively aware shigellosis, particularly vulnerable children with diarrhea, such as those younger than 5 years or identification and treatment of *Shigella* infection which might be life-saving. As a result, initiating and scaling-up drug susceptibility testing for each shigellosis case, educating the community and health care providers on appropriate antibiotic use, and conducting clinical trials are all urgently needed to support the development of management guidelines for *Shigella* infections.

## Figures and Tables

**Table 1:** Summary of 22 studies reporting the prevalence of *Shigella* and its drug resistance in East Africa, from 2015 to 2022.

Country	Authors and study population	Sample size (*N*)	Prevalence of *Shigella*	Antibiotics (%)										MDR	Conclusion
				Tetracycline (10 *µ*g)	Co-trimoxazole (125 *µ*g)	Ampicillin (30 *µ*g)	Chloramphenicol (30 *µ*g)	Gentamicin (10 *µ*g)	Ciprofloxacin (5 *µ*g)	Norfloxacin (10 *µ*g)	Nalidixic acid (30 *µ*g)	Ceftriaxone (30 *µ*g)	Amoxicillin (30 *µ*g)		
Ethiopia	Abebe et al. (2018)—among children aged below five years with diarrhea	204	17 (8.3)	—	11 (64.7)	14 (82.4)	8 (47.1)	13 (76.5)	3 (17.6)	0	0	3 (17.6)	—	11 (64.7%) (>3 antibiotics)	None of the strains were sensitive to all antimicrobials tested. Antimicrobial susceptibility testing services are required for treatment [11]
	Marami et al. (2018)—among asymptomatic food handlers	417	6 (1.4)	5 (83.3)	4 (66.7)	2 (33.3)	3 (50)	2 (33.3)	0	1 (16.7)	—	1 (16.7)		1 (10%) (>3 antibiotics)	Food handlers as potential sources of food borne infections [12]
	Lamboro et al. (2016)—outpatients visiting the hospital and had diarrhea	176	2 (1.1)	2 (100)	1 (50)	2 (100)	0	0	0	0	1 (50)	0	—	2 (100%) (>3 antibiotics)	MDR towards maximum of four drugs was observed [13]
	Mamuye et al. (2015)—among under 5 children with acute diarrhea	190	23 (9.1)	—	12 (52.2)	22 (95.7)	5 (21.7)	4 (17.4)	1 (4.3)	—	5 (21.7)	1 (4.3)	21 (91.4)	20 (87%) two up to six commonly used antibiotics)	High frequency of MDR; however, there is still a chance to use ciprofloxacin and ceftriaxone as a treatment option in the setting because of their low frequency of resistance rate [14]
	Feleke et al. (2018)—among under five children with and without diarrhea	225	5 (2.2)	—	—	5 (100)	—	—	—	—	—	0	5 (100)	—	Ceftriaxone should be considered when necessary within the context of use [15]
	Terfassa and Jida (2018)—among diarrheal patients	422	9 (2.1)	—	—	—	1 (11.1)	1 (11.1)	0	0	1 (11.1)	0	7 (77.8)	33% drugs were MDR	Resistant to most common drugs, care should be taken in selecting antimicrobials in treating disease [16]
	Mengist et al. (2018)—among food handlers in catering establishments	220	5 (2.3)	4 (80)	1 (20)	5 (100)	4 (80)	0	0	1 (20)	—	—	—	—	Tetracycline, ampicillin, and chloramphenicol should not be used for the treatment and prevention of *Shigella* species [17]
	Mama and Alemu (2016)—among food handlers	345	10 (3)	—	—	—	0	0	—	—	—	0	4 (40)	—	Constant epidemiological surveillance, improvement of personal hygiene, and environmental sanitation [18]
	Assefa and Girma (2019)——among children aged below five years with diarrhea	422	18 (4.3)	18 (100)			18 (100)		0			0	18 (100)	2 (11.1%) and 16 (88.9%) *Shigella* isolates were resistant to three and four drugs, respectively	Ciprofloxacin and ceftriaxone drugs of choice recommended for *Shigella* species [19]
	Tadesse et al. (2019)—among asymptomatic street food vendors	218	6 (2.8)	3 (50)	—	5 (83.3)	4 (66.7)	0	0		0	0	6 (100)	Higher MDR observed to ampicillin, amoxicillin, and tetracycline	Treatment requires further knowledge of the antimicrobial susceptibility pattern [20]
	Ameya et al. (2018)—among under the age of five children	167	8 (4.8)	—	2 (25)	—	4 (50)	2 (25)	0	1 (12.5)	—	—	8 (100)	Multidrug resistance was observed in majority of the isolate	Improving hygiene status of under five children and regular drug susceptibility test is important to reduce the problem [21]
	Gebrekidan et al. (2015)—among acute diarrheal outpatients	216	15 (6.9)	—	10 (66.7)	15 (100)	7 (46.7)	2 (13.3)	1 (6.7)	1 (6.7)	—	—	13 (86.7)	12 (80%) of the isolates were multidrug resistance (resistance for more than two antibiotics)	Periodic epidemiological surveillance is of great importance to control the diseases and MDR of *Shigella* species [22]
	Diriba et al. (2020)—among food handlers	220	7(3.2)	—	—	7 (100)	4 (57.1)	—	1 (14.3)	1 (14.3)	—	3 (42.9)	—	85.7% of *Shigella* isolates were recorded as MDR	Constant epidemiological surveillance and hygiene are recommended to control pathogens [23]
	Tosisa et al. (2020)—diarrheic children	239	6 (2.5)	4 (66.7)	3 (50)	5 (83.5)	2 (33.3)	0	0	—	1 (16.7)	—	3 (50)	Five *Shigella* species were MDR	A further study targeting other causes of diarrhea should be conducted to establish the major causes of childhood diarrhea in the study area [24]
	Legese et al. (2020)—among food handlers	301	11 (3.7)	—	1 (9.1)	11 (100)	2 (18.2)	1 (9.1)	0	—	—	1 (9.1)	5 (45.5)	90.9% *Shigella* species were resistant to at least three antimicrobials	Physicians should prescribe based on the laboratory result [25]
	Teshome et al. (2019)—patients with diarrhea	232	22 (9.5)	16 (72.7)	—	16 (72.7)	12 (54.5)	—	13 (59.1)	—	5 (22.7)	—	5 (22.7)	—	Treatments need to be based on species identification [26]

Kenya	Olipher et al. (2020)—patients with diarrhea	400	47 (11.8)	14 (29.8)	—	18 (39.3)	11 (23.4)	—	12 (25.5)	—	8 (17)	—	—	—	Different geographical settings have responded differently to antibiotics [27]
	Njuguna et al. (2016)—acute bloody diarrhea	284	67 (23.6)	56 (83.6)	—	39 (58.2)	14 (20.9)	—	2 (3.0)	—	3 (4.5)	0	—	Over half of the isolated *Shigella* species were MDR	There is an urgent need for a rational use of antimicrobials [28]
	Onyango et al. (2019)—from Street vended Food	186	13 (7)	0	—	0	4 (30.8)	—	—	0	0	—	0	—	More attention to food hygiene practices to eliminate the risk of spreading antibiotic-resistant pathogenic [29]

South Sudan	Mogga et al. (2015)—patients with diarrhea	286	15 (5.2)	2 (13.3)	1 (6.7)	0	1 (6.7)	4 (26.7)	0	—	1 (6.7)	0	—	—	To inform antibiotic choices, further study of antimicrobial resistance trends isneeded in the area [30]

Sudan	Moglad (2020)—from different samples sources of patients	144	1 (0.7)	—	—	1 (100)	—	—	0	0	—	—	—	MDR has increasing gradually	Proper assessments and research to manage the progress of the resistant strain [31]

Somalia	Ali Noret al. (2021)—among children aged below five years with diarrhea	180	37 (20.6)	37 (100)	37 (100)	37 (100)	—	—	8 (21.6)	—	—	16 (43.2)	—	Among the serogroups, most of the MDR phenotypes were found in *S. flexneri* (65.9%)	There is an urgent need for AMR surveillance and continuous monitoring [32]

Note: “—” means not done or did not get the information.

**Table 2:** Pooled proportions of *Shigella* prevalence and its drug resistance in East Africa from 2015 to 2022.

Antibiotics	Antibiotics resistance rates (%) reported by 22 studies					
	Total (among 350 *Shigella*isolates)	Countries				
		Ethiopia	Kenya	Sudan	South Sudan	Somalia
Tetracycline	161 (46)	52 (30.6)	70 (55.1)	—	2 (13.3)	37 (100)
Co- trimoxazole	83 (23.7)	45 (26.5)	—	—	1 (6.7)	37 (100)
Ampicillin	204 (58.3)	109 (64.1)	57 (44.9)	1 (100)	0	37 (100)
Chloramphenicol	104 (33.2)	74 (43.5)	29 (22.8)	—	1 (6.7)	—
Amoxicillin	95 (30.4)	95 (55.9)	0	—	—	—
Gentamicin	29 (9.3)	25 (14.7)	—	—	4 (26.7)	—
Ciprofloxacin	41 (11.7)	19 (11.2)	14 (11.0)	0	0	8 (21.6)
Norfloxacin	5 (1.6)	5 (2.9)	0	0	—	—
Nalidixic acid	25 (8.0)	13 (7.6)	11 (8.7)	—	1 (6.7)	—
Ceftriaxone	25 (7.1)	9 (5.3)	0	—	0	16 (43.2)
Overall prevalence of *Shigella* (350 (6.2%))		170 (4.0)	127 (14.6)	1 (0.7)	15 (5.2)	37 (20.6)

Note: “—” means not done or did not get the information.

## Data Availability

The data used to support the findings of this study are included within the manuscript.

## Abbreviations

AMR: Antimicrobial resistance
MDR: Multidrug-resistant.
